# *Anaplasma phagocytophilum* Ecotype Analysis in Cattle from Great Britain

**DOI:** 10.3390/pathogens12081029

**Published:** 2023-08-10

**Authors:** Ternenge Thaddaeus Apaa, Harriet McFadzean, Sara Gandy, Kayleigh Hansford, Jolyon Medlock, Nicholas Johnson

**Affiliations:** 1 Vector Borne Diseases, Virology Department, Animal and Plant Health Agency, Woodham Lane, Addlestone KT15 3NB, UK; 2 Animal and Plant Health Agency, Staplake Mount, Starcross, Exeter EX6 8PE, UK; 3 Medical Entomology & Zoonoses, UK Health Security Agency, Porton Down, Salisbury SP4 0JG, UK

**Keywords:** livestock, tick-borne fever, *Ixodes ricinus*, ecotypes

## Abstract

*Anaplasma phagocytophilum* (*A. phagocytophilum*) is the aetiological agent of tick-borne fever in cattle and sheep, and granulocytic anaplasmosis in human and dogs. Livestock, companion animal and human infections with *A. phagocytophilum* have been reported globally. Across England and Wales, two isolates (called ecotypes) have been reported in ticks. This study examined *A. phagocytophilum* isolates present in livestock and wildlife in Great Britain (GB), with a particular focus on cattle. Clinical submissions (EDTA blood) from cattle (*n* = 21) and sheep (*n* = 3) were received by APHA for tick-borne disease testing and the animals were confirmed to be infected with *A. phagocytophilum* using a PCR targeting the *Msp*2 gene. Further submissions from roe deer (*n* = 2), red deer (*n* = 2) and *Ixodes ricinus* ticks (*n* = 22) were also shown to be infected with *A. phagocytophilum*. Subsequent analysis using a nested PCR targeting the *groEL* gene and sequencing confirmed the presence of ecotype I in cattle, sheep, red deer and *Ixodes ricinus*, and ecotype II in roe deer and *I. ricinus* removed from deer carcasses. Despite the presence of two ecotypes, widely distributed in ticks from England and Wales, only ecotype I was detected in cattle in this study.

## 1. Introduction

*Anaplasma phagocytophilum* is an obligate intracytoplasmic bacteria classified under the family *Anaplasmatacea* (formerly *Ehrlichia equi* and *Ehrlichia phagocytophila*). It was renamed *A. phagocytophilum* in 2001 based on a genetic analysis unifying the former species *Ehrlichia equi*, *Ehrlichia phagocytophila* and the agent of human granulocytic ehrlichiosis [1,2]. *A. phagocytophilum* is transmitted by ticks and has been reported in cattle (*Bos taurus*), sheep (*Ovis aries*), goat (*Capra hircus*), horse (*Equus caballus*), dog (*Canis lupus familiaris*), red deer (*Cervus elaphus*), roe deer (*Capreolus capreolus*), white-tailed deer (*Odocoileus virginianus*), rodents, birds, hedgehogs (*Erinaceus europaeus*), wild boar (*Sus scrofa*), humans, cat (*Felis catus*), red fox (*Vulpes vulpes*) and moose (*Alces alces*). *Ixodes ricinus* is the principal vector of *A. phagocytophilum* in Europe [3,4], although its DNA has also been detected from *Dermacentor reticulatus* [5], *D. marginatus* [6], *Haemaphysalis punctata* [7], *H. concinna* [5] and other *Ixodes* species, including *I. hexagonus*, *I. canisuga* [8], *I. persulcatus* and *I. trianguliceps* [9]. However, the role of these tick species as potential vectors for *A. phagocytophilum* transmission in Europe is not clearly understood. *Ixodes scapularis* and *I. pacificus* transmit *A. phagocytophilum* in North America, where human granulocytic anaplasmosis is regularly reported [10,11,12], although there appears to be few reports of infection in livestock. In Asia and Russia, *I. persulcatus* and *Dermacentor silvarum* are known vectors of *A. phagocytophilum* [13]. Transmission from mother to child immediately before or after birth has been documented in humans [14]; transmission to the foetus via placenta has also been reported in cattle [15]. Cases of canine, ovine, equine, bovine and human anaplasmosis have been reported globally and the infection of questing ticks, ruminants and wildlife has been reported in Europe over the past decade [16,17,18,19,20].

Human infection with *A. phagocytophilum* is considered an emerging zoonotic disease. It was first recorded as a febrile illness and named human granulocytic ehrlichiosis (HGE) in North America in 1994 [21]. Case rates have dramatically increased since, and the disease poses an increasingly important public health threat. The first European cases were documented in Slovenia [22,23,24,25], with further cases reported in other European countries, including Spain [4,26], France [27] and Italy [28]. However, overall disease rates remain substantially lower in Europe than in the United States. The rarity of human infection with *A. phagocytophilum* in GB has been considered to be due to the absence of zoonotic isolates, also described as ecotypes [18]. Recent studies have documented *A. phagocytophilum* presence in GB, including a prevalence survey of questing ticks in Wales (12.1%), Northern England (4.7%), Southern England (1.8%) and central England (0.5%) [17]. Ecotype I has been recorded in GB sheep (*O. aries*) [18] associated with specific areas and is considered more likely to infect humans [17]. Gandy et al. [17] and Bianchessi et al. [18] examined questing ticks and sheep, respectively. However, cases of bovine anaplasmosis [29,30] and co-infection with *Babesia* and *A. phagocytophilum* resulting in severe disease have been recorded in GB and Europe [16,31,32]. Bovine anaplasmosis is a transient and mild infection characterized by clinical signs such as fever, anorexia, weakness and secondary infection due to immunosuppression [33]. High morbidity associated with abortion and a drop in milk production, resulting in economic losses, have also been reported in dairy cattle diagnosed with bovine anaplasmosis [32,33,34]. However, information regarding the potential role of *A. phagocytophilum* in the pathogenesis and diagnosis of abortions and stillbirth in cattle is not clearly known [34].

To establish the evolutionary relationship of *Anaplasma* species, a selection of appropriate molecular gene marker is essential. PCR assays designed to amplify *16S rRNA* [13], highly conserved major surface protein-2 (*Msp2* or *p44*), citrate synthase (*gltA*) [35,36], *ankA* [37] and heat shock protein (*groEL*) [17,18] genes have all proven to be useful. In addition, PCR and sequencing assays designed to target multiple genes (multilocus sequence genotyping) have been used effectively for *A. phagocytophilum* genotyping [37,38]. However, the *groE*L gene has demonstrated to be a valuable and useful genetic marker to identify and establish distinct genetic clades of *A. phagocytophilum* in the absence of multilocus sequence genotyping [18,39,40]. Information on *A. phagocytophilum* ecotypes present in GB cattle is currently unavailable. The aim of this study was to investigate *A. phagocytophilum* isolates present in GB cattle from samples (initially tested using *Msp2* gene qPCR) obtained from Southern England, Wales and Scotland. Additional samples obtained from sheep, deer and *I. ricinus* were also examined.

## 2. Materials and Methods

### 2.1. Sample Collection, Polymerase Chain Reaction (PCR) and Sequencing

Whole engorged ticks (collected from cattle and roe deer only) and EDTA blood samples collected from cattle (*Bos taurus*), sheep (*O. aries*) and both roe (*Capreolus capreolus*), and red (*Cervus elaphus*) deer submitted from nine locations (for tick-borne pathogen testing) in Great Britain, including England (Devon, Cornwall, Norfolk, Cumbria, Somerset, and Dorset), Scotland and Wales (Pembrokeshire and Powys), were examined. Samples were tested for *A. phagocytophilum* following DNA extraction using QIAamp DNeasy kit (QIAgen, Manchester, UK). *Msp2* gene-specific TaqMan qPCR assay [41] was used to initially screen samples for the presence of *A. phagocytophilum* DNA. Where this was detected, a partial *groEL* gene heminested PCR assay was applied [39]. PCR products obtained were separated on agarose gel (1.5%) prepared with SYBR safe nucleic acid staining dye (Thermo Fisher Scientific, UK) and run at 100 volts for 60 min. PCR controls consisted of *A. phagocytophilum* DNA (positive) [16] and nuclease-free water (negative). The g*roEL* gene PCR products were sequenced using primers Ephpl *groEL* (569) and Eph *groEL* (1142). Morphological identification and molecular barcoding of tick samples were carried out, following published methods [42]. Details of all PCR primers, master mix reaction volumes and thermal cycling conditions are provided in the Supplementary file (Supplementary Materials Table S4).

### 2.2. Sequence Editing, Assembly and Phylogenetic Analysis

Quality assessment, removal of primer sequence, editing, alignment and generation of consensus sequence from Sanger sequence obtained was carried out using SeqMan Pro in DNAStar Lasergene v15, followed by NCBI online BLASTn search. *A*. *phagocytophilum* g*roEL* gene DNA sequences generated were deposited in NCBI GenBank under accession number OQ436965-OQ437012. A total of 328 *A. phagocytophilum groEL* gene nucleotide sequences, including sequences generated from this study, downloaded from GenBank database and published in the recent literature [18,43,44], were used for phylogenetic analysis. A maximum likelihood phylogenetic tree was constructed in IQ-TREE v2.0.7 [45], using 10,000 bootstrap approximations and implementation of UFBoot2 within IQ-TREE to evaluate nodes [46]. IQ-TREE v2.0.7 automatically selected the best nucleotide substitution model (HKY+F+R2) [47] with the lowest Bayesian information criterion (BIC) [48]. The precision of the phylogenetic tree generated was improved by the inclusion of the ModelFinder option in command-line for the selection of the best fitting nucleotide substitution model [48]. The tree constructed was visualized and annotated in FigTree v1.4.4. to produce a radial phylogram for simple presentation.

## 3. Results

**Figure 1:**
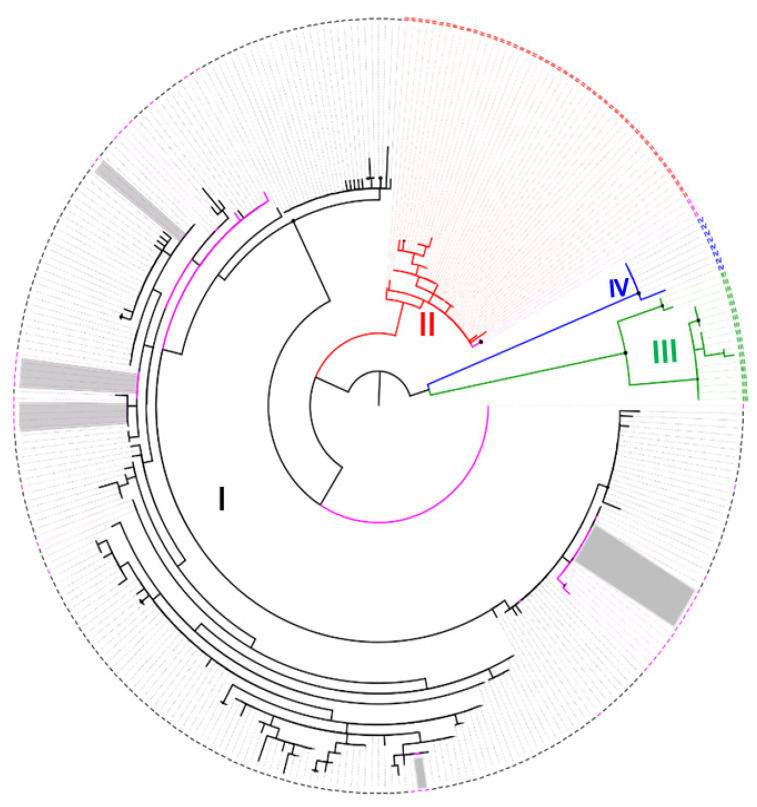
A simplified radial phylogram displayed following a maximum likelihood phylogenetic tree constructed with 10,000 bootstrap approximation using *A. phagocytophilum groEL* gene sequences generated from this study (*n* = 48) and representative ecotypes (*n* = 328) from previous studies [18,43]. Clades, branches and branch tips in black represents ecotype I, red represents ecotype II, green represents ecotype III and blue represents ecotype IV, while taxa in magenta under ecotypes I and II represent nucleotide sequences reported from this study. Branch tips highlighted in grey under ecotype I clade represents *A. phagocytophilum* ecotype I sequences reported from Great Britain cattle. Roman numerals I, II, III and IV represents ecotypes. The phylogeny is rooted at midpoint and drawn to display circular node shapes at bootstrap support values ≥ 90.

A total of fifty samples that previously tested positive for the presence of *A. phagocytophilum* via the *Msp2* gene qPCR assay were assessed for *A. phagocytophilum* genotyping, following *groEL* gene PCR assay. All tick samples assessed (*n* = 22; female: 20; nymphs: 2) were identified as *I. ricinus*. *A. phagocytophilum groEL* gene sequence was obtained for 48 of 50 samples tested, including 20/22 *I. ricinus*, 21/21 *Bos taurus*, 3/3 *O. aries*, 2/2 *Capreolus capreolus* and 2/2 *Cervus elaphus* samples (Table 1). Nucleotide sequences (530 bp) were assembled from confirmed positive samples (48/48). NCBI online custom BLASTn search demonstrated 95–100% a similarity between sequences generated by this study and *A. phagocytophilum* sequences submitted from the British Isles and Europe (Supplementary Materials Table S2). Although all nucleotide sequences obtained were >90% identical to other *A. phagocytophilum* sequences available in the GenBank database, 17/48 of sequences obtained were >99% identical to sequences (accession numbers: KJ832487, KJ832484) originating from *Bos taurus* (France) and *O. aries* (AF548385) from Norway. Maximum likelihood phylogenetic analysis demonstrated that 45/48 sequences generated in this study clustered with *A. phagocytophilum* sequences identified as ecotype I, while the remaining three (3/48) clustered with ecotype II (Figure 1, Supplementary Materials Figure S1 [18]. This set of three *A. phagocytophilum* sequences were generated from *C. capreolus* blood/tick samples and showed a greater identity (NCBI online BLASTn search) to *A. phagocytophilum* sequences (accession number KM215256, JN005745, DQ779568 and AF383226) detected from *C. capreolus* from Poland and Switzerland, classified as ecotype II in the recent literature [43]. In addition, phylogenetic tree demonstrated a geographical clustering of *A. phagocytophilum* ecotype I reported in this study (from sheep (1/3), cattle (8/21) and *I. ricinus* (8/20)) with previously reported European ecotype I from cattle, humans (e.g., AF033101: Austria; AF033101: Slovenia), horses, hedgehog, brown bear, polecat, sheep, roe deer, dogs, wild goat, fox and dogs. Similarly, the remaining ecotype I sequence obtained from Great Britain, cattle (*n* = 13), sheep (*n* = 2) and ticks (*n* = 10), in this study, clustered with previously reported European ecotype I from wild ruminants, cattle, sheep, horse and dog. However, the American ecotype I reference sequences reported from domestic and wild animals (horses, rodents, rabbits, dogs and cats) and humans (zoonotic ecotype I) clustered within a distinct geographical subclade (Supplementary Materials Figure S1).

## 4. Discussion

Following the renaming of *A. phagocytophilum* [1], genotypes with a diverse host range have emerged. The emergence of multiple genotypes has created complexity in the understanding of pathogenicity and clinical disease manifestation in various animal hosts and potential for zoonotic transmission in humans. Bovine anaplasmosis caused by *A. phagocytophilum* is associated with a severe clinical disease in cattle globally, and risk factors such as the season of the year, the presence of tick vectors, latitude and farm hygiene practice have been associated with disease [49]. However, this study aimed at establishing *A. phagocytophilum* ecotypes using a small number of samples confirmed to be positive via the *Msp2* qPCR. To the best of our knowledge, this is the first study to demonstrate the presence of ecotype I in cattle and red deer in GB. Bovine anaplasmosis and co-infection with *B. divergens* and *A. phagocytophilum* characterized by severe disease and mortality has been reported in GB [16,32] and other parts of mainland Europe [29,30,31]. Although this study did not test for *Babesia* species, *B. divergens* is known to share the same vector (*I. ricinus*) as *A. phagocytophilum* and co-infection can increase the severity of disease [16]. *I. ricinus* is a three-host tick, both larval and nymphal stages of *I. ricinus* feed on small to medium-sized animals while adults prefer larger animals, including cattle. The presence of *A. phagocytophilum* (ecotype I) in all engorged ticks collected from cattle is likely to originate from a previous blood meal (during larval/nymphal lifecycle stages from other animal hosts) or present in the blood meal obtained from infested cattle. This report supports previous findings that infected animals can play a significant role in the enzootic transmission of *A. phagocytophilum* [50]. Additional studies are required to provide a clear understanding of the pathogenicity and clinical manifestation of *A. phagocytophilum* in cattle, and assist clinicians in diagnosis, treatment and control. The presence of ecotype I in sheep and both ecotypes (I and II) in *I. ricinus* further supports recent studies reporting *A. phagocytophilum* in sheep [18] and questing ticks collected from GB recreational areas [17], while the report of ecotype II from roe deer in this study supports previous reports from Central Europe [51,52]. Cervids (red and roe deer) are wildlife reservoir hosts for various genotypes of *A. phagocytophilum* known to be transmitted by *I. ricinus* [53,54,55]. Although roe deer have been demonstrated to be co-infected with multiple *A. phagocytophilum* variants, these variants are not considered pathogenic to domestic animals and humans. However, genotypes reported from red deer are pathogenic to domestic animals [51]. The presence of ecotype I in red deer from this study suggest further investigations are needed to understand the role of red deer in *A. phagocytophilum* transmission in GB. In addition, the geographical clustering of ecotype I sequences reported in this study partly supports previous findings in Europe [56]. Ecotype I reported in this study clustered within the same geographical subclade with European wild and domestic animals, equine and human zoonotic ecotypes I sequences, suggesting zoonotic risk and the likelihood of human infection as previously described. The separate geographical clustering of the American human, domestic and wild animal ecotype I may likely suggest differences in potentials for zoonotic infections between the European and American ecotype I as recently described [18]. This report provides additional information on the *A. phagocytophilum* isolates infecting cattle in GB.

## 5. Conclusions

In conclusion, this study reports that only *A. phagocytophilum* ecotype I has been detected in GB cattle and provides further evidence for the presence of ecotypes I and II in red and roe deer, respectively. The presence of ecotype I in cattle and *I. ricinus* infesting cattle corroborates such reports in previous studies. These findings constitute the first report of *A. phagocytophilum* ecotype I in GB cattle, despite the presence of ecotypes I and II in the indigenous tick population [17]. However, this report should be interpreted with caution owing to the smaller sample size assessed in this study. The presence of *A. phagocytophilum* ecotype I in sheep was also confirmed in this study as previously reported in GB [18]. The epidemiology and clinical disease manifestation of the *A. phagocytophilum* genotypes in GB cattle require further investigation as *A. phagocytophilum* and/or co-infection with *B. divergens* in cattle is routinely reported and likely to result in severe disease and potential mortality. While the full zoonotic potentials of ecotype I is not well understood, it has been associated with human granulocytic anaplasmosis. Preventive measures, including public education on tick control and limiting the exposure of animal and humans to tick bites, are suggested measures in the absence of adequate information on *A. phagocytophilum* disease pathogenicity and epidemiology in GB cattle.

## Figures and Tables

**Table 1:** Distribution of *A. phagocytophilum* ecotypes among GB cattle, sheep, roe/red deer and ticks in Great Britain.

Location	Ecotype	Sequence Variants (n)	Host	Tick Species	Negative (n)	Positive (n)	Total
Devon	1	8	*B. taurus*		−	8	8
	1	13	vegetation	*I. ricinus* *	2	13	15
Cornwall	1	2		*I. ricinus* *	−	2	2
	1	5	*B. taurus*		−	5	5
Norfolk	1	2	*C. elaphus* (red deer)		−	2	2
Cumbria	1	1		*I. ricinus* *	−	1	1
	2	1	*C. capreolus* (roe deer)		−	1	1
Pembrokeshire	1	2		*I. ricinus* *	−	2	2
Dorset	1	1	*O. aries*		−	1	1
	1	1	*B. taurus*		−	1	1
Somerset	1	2	*B. taurus*		−	2	2
	1	1	*C. capreolus* (roe deer)		−	1	1
Powys	1	1	*B. taurus*		−	1	1
Scotland	1	1	*O. aries*		−	1	1
Unknown	2	2		*I. ricinus* **	−	2	2
	1	5	*B. taurus*		−	4	4
	1	1	*O. aries*		−	1	2
Total		48			2	48	50

(*) Ticks collected from cattle; (**) ticks collected from roe deer carcass. NB: Only engorged ticks were examined.

## Data Availability

Nucleotide sequences obtained from this study were deposited in the NCBI GenBank under accession number OQ436965-OQ437012 (Supplementary Materials Table S1).

## Supplementary Materials

The following supporting information can be downloaded at: https://www.mdpi.com/article/10.3390/pathogens12081029/s1. Figure S1: A full polar cladogram displayed, following maximum likelihood phylogenetic tree constructed at 10,000 bootstrap approximations using A. phagocytophilum groEL gene nucleotide sequences (48) generated from this study and representative ecotypes (328) reported in the previous literature; Table S1: GenBank accession number and list of A. phagocytophilum nucleotide sequences obtained from GB livestock, I. ricinus and deer species; Table S2: A summary of NCBI online custom BLASTn search containing a list of GB nucleotide sequences obtained from GB livestock, I. ricinus and deer species, GenBank accession numbers and percentage identity, established ecotypes based on the recent literature, host and country of origin; Table S3: List of GenBank nucleotide accession numbers used for NCBI online BLASTn search; Table S4: List of published nested PCR primers (A), reaction volumes and thermal cycling conditions (B) used for the amplification of the A. phagocytophilum groEL gene in this study. Available online: https://zenodo.org/record/8174242 (accessed on 15 July 2023).
