# Supplementary material for: Anaplasma phagocytophilum Ecotype Analysis in Cattle from Great Britain
Source: Pathogens. 2023 Aug 10;12(8):1029. doi: 10.3390/pathogens12081029 (PMC10459425; doi:10.3390/pathogens12081029)
Supplement: Supplementary file 1 [file pathogens-12-01029-s001.zip › pathogens-2513457-supplementary.pdf]

Supplementary Material

List of supplementary tables

Table S1: GenBank accession number and list of *A. phagocytophilum* nucleotide sequences obtained from GB livestock, *I. ricinus* and deer species; Table S2: A summary of NCBI online custom BLASTn search containing list of GB nucleotide sequences obtained from GB livestock, *I. ricinus* and deer species, GenBank accession numbers and percentage identity, established ecotypes based on recent literature, host and country of origin; Table S3: List of GenBank nucleotide accession numbers used for NCBI online BLASTn search; Table S4: List of published nested PCR primers (A), reaction volumes and thermal cycling conditions (B) used for the amplification *A. phagocytophilum groEL* gene in this study.

Additional file 2: Table S1. GenBank accession number and list of *Anaplasma phagocyto philum* nucleotide sequences obtained from GB livestock, *I. ricinus* and deer species.

**Sequence_ID**	**Accession_number**
A.pha./f/I.ric-M13.2/GB	OQ436965
A.pha./f/I.ric-M13.3/GB	OQ436966
A.pha./f/I.ric-Pem-6-2021/GB	OQ436967
A.pha./f/I.ric-Pem-7-2021/GB	OQ436968
A.pha./h1/B.tau-Dev-2021/GB	OQ436969
A.pha./h2/B.tau-Dev-2021/GB	OQ436970
A.pha./n1/I.ric-Cor-2021/GB	OQ436971
A.pha./n2/I.ric-Cor-2021/GB	OQ436972
A.pha. /O.ar-100857-Dor-2021/GB	OQ436973
A.pha/B.tau-C315970-2023/GB	OQ436974
A.pha/B.tau-C316672-2023/GB	OQ436975
A.pha/B.tau-C316675-2023/GB	OQ436976
A.pha/C.cap-M0404-2023/GB	OQ436977
A.pha/C.cap-M413-2021/GB	OQ436978
A.pha/C._ela-Nor405-2021/GB	OQ436979
A.pha/C.ela-Nor408-2021/GB	OQ436980
A.pha/f.I.ric/n=5/Cod-29-Dev-2021/GB	OQ436981
A.pha/f/I.ric/n_5/Cod-5-Dev-2021/GB	OQ436982
A.pha/f/B.tau-32b-2023/GB	OQ436983
A.pha/f/B.tau-089-2023/GB	OQ436984
A.pha/f/B.tau-103-Cor-2021/GB	OQ436985
A.pha/f/B.tau-169b-2023/GB	OQ436986
A.pha/f/B.tau-229b-2023/GB	OQ436987
A.pha/f/B.tau-255-2023/GB	OQ436988
A.pha/f/B.tau-292d-2023/GB	OQ436989
A.pha/f/B.tau-308-2023/GB	OQ436990
A.pha/f/b.tau-309b-2023/GB	OQ436991
A.pha/f/b.tau-329-2023/GB	OQ436992
A.pha/f/B.tau-343b-2023/GB	OQ436993
A.pha/f/B.tau-343c-2023/GB	OQ436994
A.pha/f/B.tau-483-2021/GB	OQ436995
A.pha/f/B.tau-316672-Dev-2021/GB	OQ436996
A.pha/f/B.tau-317934-2023/GB	OQ436997
A.pha/f/I.ric/BoWh-18-Cum-2021/GB	OQ436998
A.pha/f/I.ric/n_5/Cod-5-Dev-2021/GB	OQ436999
A.pha/f/I.ric/n_5/Cod-7-Dev-2021/GB	OQ437000
A.pha/f/I.ric/n=5/Cod-32-Dev-2021/GB	OQ437001
A.pha/f/I.ric-Dar-21-Devon-2021/GB	OQ437002
A.pha/f/I.ric-Exm15-Dev-2021/GB	OQ437003
A.pha/m/I.ric/n=5/Cod-4-Dev-2021/GB	OQ437004
A.pha/n/I.ric/n=10/Cod-9-Dev-2021/GB	OQ437005
A.pha/n/I.ric/n=10/Cod-12-Dev-2021/GB	OQ437006
A.pha/n/I.ric/n=10/Cod-17-Dev-2021/GB	OQ437007

Sequence_ID	Accession_number
A.pha/n/I.ric/n=10/Cod-23-Dev-2021/GB	OQ437008
A.pha/n/I.ric/n=10/Cod-26-Dev-2021/GB	OQ437009
A.pha/n/I.ric/n_10/Cod-28-Dev-2021/GB	OQ437010
A.pha/O.ari-S029-2023/GB	OQ437011
A.phag/O.ari-S03401-2023/GB	OQ437012

Nucleotide sequences obtained from this study were name using pathogen (*A. phagocytophilum*) name, sequence source/host and the year samples were collected etc. as below: A.pha. (*Anaplasma phagocytphilum*); h1 or h2.B.tau (heifer1 or 2, *Bos taurus*); f.B.tau (female, *Bos taurus*); f.I.ric (female, *Ixodes ricinus*), O.ari (*Ovis aries*); n1 or n2/I.ric (nymph, *Ixodes ricinus*); C.ela. (*Cervus elaphus*); n = x means pooled samples, C.cap (*Capreolus capreolus*).

Additional file 3: Table S2: A summary of NCBI online custom BLASTn search containing list of nucleotide sequences obtained from GB livestock, *I. ricinus* and deer species, GenBank accession numbers and percentage identity, established ecotypes based on recent literature, host and country of origin.

**Sequence Id**	**Accession numbers matched**	**No of sequences matched**	**Ecotype**	**Accession host/country**	**Percentage (%) identity**
A.pha./f/I.ric-M13.2/GBseq14-A.pha/C.cap-M413-2021/GB<br>A.pha./f/I.ric-M13.3/GBseq13-A.pha/C.cap-M0404-2023/GB	KM215256 (all 4 matched)	4	II	*Capreolus capreolus*/Slovenia	99.8
	JN005745		II	*Capreolus capreolus*/Poland	
	DQ779568		II	*Capreolus capreolus*/Poland	
	AF383226		II	*Capreolus capreolus*/Switzerland	
A.pha./f/I.ric-Pem-6-2021/GB<br>A.pha/C.ela-Nor405-2021/GB	KJ832471	2	I	*Equus caballus*/France	99.8
	U96729		I	*Capreolus capreolus*/GB	
A.pha./f/I.ric-Pem-7-2021/GB<br>A.pha/f/B.tau-316672-Dev-2021/GB<br>A.pha/f/I.ric-Dar-21-Devon-2021/GB<br>A.pha/f/I.ric-Exm15-Dev-2021/GB	KJ832476	4	I	*Bos taurus*/France	100
A.pha./h1/B.tau-Dev-2021/GB<br>A.pha./n1/I.ric-Cor-2021/GB<br>A.pha./n2/I.ric-Cor-2021/GB<br>A.pha/f/I.ric/n=5/Cod-32-Dev-2021/GB<br>A.pha/f/B.tau-103-Cor-2021/GB<br>A.pha/f/B.tau-169b-2023/GB<br>A.pha/f/B.tau-229b-2023/GB<br>A.pha/f/B.tau-255-2023/GB	KJ832478	8	I	*Bos taurus*/France	100
A.pha./O.ar-100857-Dor-2021/GB<br>A.pha/C.ela-Nor408-2021/GB	MG670108 (identical to both)	2	I	*Capreolus capreolus*/Poland	99.8
	KJ832473		I	*Bos taurus*/France	
.pha/B.tau-C315970-2023/GB<br>A.pha/O.ari-S029-2023/GB	KJ832474	2	I	*Bos taurus*/France	100
A.pha/B.tau-C316672-2023/GB	KM215264	1	I	*Capreolus capreolus*/Slovenia	99.8
	U96730		I	*Capreolus capreolus*/GB	
A.pha/B.tau-C316675-2023/GB	KJ622307	1	I	*Ursus arctos*/Slovenia	99.8
A.pha/f.I.ric/n=5/Cod-29-Dev-2021/GB<br>A.pha/f/B.tau-329-2023/GB	KJ832483	2	I	*Bos taurus*/France	100
A.pha/f/B.tau-089-2023/GB<br>A.pha/f/B.tau-317934-2023/GB	AF478552	2	I	*Cervus elaphus* Slovenia	100
A.pha./h2/B.tau-Dev-2021/GBseq18-013b-2023-GB<br>A.pha/f/B.tau-32b-2023/GB<br>A.pha/f/B.tau-292d-2023/GB	KJ832487/<br>KJ832484	17	I	*Bos taurus*/France	100
	AF548385		I	*Ovis aries*/Norway	

**Sequence Id**	**Accession numbers matched**	**No of sequences matched**	**Ecotype**	**Accession host/country**	**Percentage (%) identity**
A.pha/f/B.tau-308-2023/GB<br>A.pha/f/B.tau-343b-2023/GB<br>A.pha/f/B.tau-343c-2023/GB<br>A.pha/f/I.ric/BoWh-18-Cum-2021/GB<br>A.pha/f/I.ric/n_5/Cod-5-Dev-2021/GB<br>A.pha/f/I.ric/n_5/Cod-7-Dev-2021/GB<br>A.pha/m/I.ric/n=5/Cod-4-Dev-2021/GB<br>A.pha/n/I.ric/n=10/Cod-9-Dev-2021/GB<br>A.pha/n/I.ric/n=10/Cod-23-Dev-2021/GB<br>A.pha/n/I.ric/n=10/Cod-26-Dev-2021/GB<br>A.pha/n/I.ric/n_10/Cod-28-Dev-2021/GB					
A.pha/f/b.tau-309b-2023/GB	MF061233	1	I	*Capreolus capreolus*/Slovenia	99.9
	JN656296		I	*Canis familiaris/*Finland	
A.pha/f/B.tau-483-2021/GB	KJ832463	1	I	*Capreolus capreolus*/France	98.9
	GQ452227		I	*Capra aegagrus*/Switzerland	
A.phag/O.ari-S03401-2023/GB	U96735	1	I	*Capreolus capreolus* Switzerland	100
Total		48			

Additional file 4: Table S3. List of GenBank nucleotide accession numbers used for NCBI online BLASTn search.

AB454079,AF033101,AF172158,AF192796,AF383226,AF478552,AF482760,AF548385,AY219849,AY220467,AY529489,AY848747,DQ088133,DQ680012,DQ779568,EF647585,EU157921,EU839853,EU860087,EU982549,GQ452225,GQ452227,GQ988754,GQ988755,GQ988774,HM057223,HM057224,HQ630615,HQ630617,JF494833,JF494834,JF893915,JN005744,JN005745,JN055359,JN055360,JN656296,JN935930,KC583431,KC753762,KC800984,KF015601,KF031385,KF383228,KF383230,KF569921,KF569925,KF745743,KF745744,KF836094,KJ622307,KJ677107,KJ832449,KJ832450,KJ832462,KJ832463,KJ832464,KJ832468,KJ832469,KJ832470,KJ832471,KJ832473,KJ832474,KJ832475,KJ832476,KJ832478,KJ832480,KJ832481,KJ832482,KJ832483,KJ832484,KJ832485,KJ832487,KM215253,KM215254,KM215256,KM215257,KM215258,KM215259,KM215260,KM215261,KM215262,KM215263,KM215264,KM215265,KM215266,KR092132,KT192430,KT220191,KT220192,KT220193,KT970678,KT970679,KT970680,KU519284,KU519285,KU519286,KU712098,KU712104,KU712105,KU712108,KU712117,KU712127,KU863662,KY379956,LC334016,MF061233,MG570466,MG670108,U96727,U96728,U96729,U96730,U96735,EU157920,KU712123,HQ630619

Additional file 5: Table S4. List of published nested PCR primers (A), reaction volumes and thermal cycling conditions (B) used for the amplification *Anaplasma phagocytophilum groEL* gene in this study (Alberti et al., 2005)

A

**PCR round**	**Primer name**	**Nucleotide sequence**	**Product length (kb)**	**Annealing temperature (AT)**
**First**	Ephpl *groEL* (569)	5'-ATGGTATGCAGTTTGATCGC-3'	624 bp	56 °C
	Ephpl *groEL* (1193)	5'-TCTACTCTGTCTTTGCGTTC-3'		
**Second**	Ephpl *groEL* (569)	5'-ATGGTATGCAGTTTGATCGC-3'	530 bp	60 °C
	Ephl *groEL* (1142)	5'-TTGAGTACAGCAACACCACCGGAA-3'		

B

***Anaplasma phagocytophilum* PCR mix**

Reagents	25 µl X1	Final conc.
Nuclease free water	8.5 µl	
2x iTaq universal Sybr mix	12.5 µl	
Forward primer	1 µl	0.4 µM
Reverse primer	1 µl	0.4 µM
Subtotal	23 µl	
Template DNA	2 µl	
Total reaction volume	25 µl	

Nested *Anaplasma Phagocytophilum* PCR thermal cycling conditions

PCR cycle	Temperature	Time
• Initial denaturation	94 °C	10 min
Follow by 40 cycles:		
• Denaturation	94 °C	30 s
• Annealing (start)	AT	30 s
• Extension	72 °C	1 min
Followed by:		
• Final extension	72 °C	5 min
• End temperature	4 °C	

Abbreviations

A. phagocytophilum: *Anaplasma phagocytophilum*; B.tau: *Bos taurus*; I.ric: *Ixodes ricinus*; O.ari: *Ovis aries*; C.ela: *Cervus elaphus*; C.cap: *Capreolus capreolus*; EDTA: Ethylenediamine tetra acetic acid (blood anticoagulant); GB: Great Britain; B.tau: *Bos taurus*
